# Functional Differences of Very-Low-Density Lipoprotein Receptor Splice Variants in Regulating Wnt Signaling

**DOI:** 10.1128/MCB.00235-16

**Published:** 2016-09-26

**Authors:** Qian Chen, Yusuke Takahashi, Kazuhiro Oka, Jian-xing Ma

**Affiliations:** a Department of Physiology, The University of Oklahoma Health Sciences Center, Oklahoma City, Oklahoma, USA; b Department of Medicine, The University of Oklahoma Health Sciences Center, Oklahoma City, Oklahoma, USA; c Harold Hamm Diabetes Center, The University of Oklahoma Health Sciences Center, Oklahoma City, Oklahoma, USA; d Department of Molecular and Cellular Biology, Baylor College of Medicine, Houston, Texas, USA

## Abstract

The very-low-density lipoprotein receptor (VLDLR) negatively regulates Wnt signaling. VLDLR has two major alternative splice variants, VLDLRI and VLDLRII, but their biological significance and distinction are unknown. Here we found that most tissues expressed both VLDLRI and VLDLRII, while the retina expressed only VLDLRII. The shed soluble VLDLR extracellular domain (sVLDLR-N) was detected in the conditioned medium of retinal pigment epithelial cells, interphotoreceptor matrix, and mouse plasma, indicating that ectodomain shedding of VLDLR occurs endogenously. VLDLRII displayed a higher ectodomain shedding rate and a more potent inhibitory effect on Wnt signaling than VLDLRI *in vitro* and *in vivo*. O-glycosylation, which is present in VLDLRI but not VLDLRII, determined the differential ectodomain shedding rates. Moreover, the release of sVLDLR-N was inhibited by a metalloproteinase inhibitor, TAPI-1, while it was promoted by phorbol 12-myristate 13-acetate (PMA). In addition, sVLDLR-N shedding was suppressed under hypoxia. Further, plasma levels of sVLDLR-N were reduced in both type 1 and type 2 diabetic mouse models. We concluded that VLDLRI and VLDLRII had differential roles in regulating Wnt signaling and that decreased plasma levels of sVLDLR-N may contribute to Wnt signaling activation in diabetic complications. Our study reveals a novel mechanism for intercellular regulation of Wnt signaling through VLDLR ectodomain shedding.

## INTRODUCTION

The canonical Wnt pathway is an evolutionarily conserved signaling pathway that regulates cell-to-cell interactions during embryogenesis and adult tissue hemostasis (1, 2). Dysregulation of Wnt signaling plays pathogenic roles in numerous diseases, such as cancer, hypertensive cardiomyopathy, diabetic retinopathy, and diabetic nephropathy (3–6). The very-low-density lipoprotein receptor (VLDLR) is a transmembrane receptor in the low-density lipoprotein receptor (LDLR) family (7). VLDLR binds a variety of ligands, such as lipoproteins, proteinases, proteinase-inhibitor complexes, vitamins, reelin, and other macromolecules, to mediate lipid metabolism and other biological functions (8–10). Previously, we identified that VLDLR functions as a negative regulator of canonical Wnt signaling (11). *VLDLR*^−/−^ mice are viable and fertile (12) but display abnormal intraretinal and subretinal neovascularization, indicating that VLDLR plays a critical role in retinal angiogenesis (13–15). In the retina of *VLDLR*^−/−^ mice, Wnt signaling is overactivated, subsequently promoting the production of vascular endothelial growth factor (VEGF) and inflammatory factors and ultimately resulting in pathological angiogenesis and inflammation in the retina (11). VLDLR forms a heterodimer with a Wnt coreceptor, LDLR-related protein 6 (LRP6), through its extracellular domain, resulting in internalization and degradation of LRP6 and, consequently, Wnt signaling inhibition (16).

VLDLR has two major variants, VLDLRI and VLDLRII, which are derived from alternative mRNA splicing. VLDLRI is the full-length variant, while VLDLRII lacks an O-linked sugar domain encoded by exon 16 (17, 18). VLDLRI is highly expressed in the heart, muscle, and adipose tissue, while VLDLRII predominates in nonmuscle tissues, including cerebrum, kidney, spleen, adrenal gland, testis, ovary, and uterus (17). However, it remains unclear why VLDLR is differentially spliced in different tissues and whether the VLDLR splice variants have differential biological functions. Studies have shown that VLDLRI and VLDLRII have different affinities for β-VLDL binding (17). It has also been reported that VLDLRI and VLDLRII are differentially expressed in cancer cell lines with various differentiations (19, 20). These studies suggest that the VLDLR splice variants may have different functions in tissues.

The shed soluble extracellular domain of integral membrane proteins are shown to have biological functions. For instance, shed soluble intercellular adhesion molecule 1 (ICAM-1) binds lymphocyte function-associated antigen 1 and blocks rhinovirus infection (21, 22). The shed form of VEGF receptor-1 is a naturally occurring inhibitor of angiogenesis (23). Recently, using a forced expression system, studies have shown that VLDLRII undergoes rapid extracellular domain shedding, releasing its soluble N-terminal extracellular fragment into the culture medium (24). In addition, we have demonstrated that the shed soluble extracellular domain of recombinant VLDLR (sVLDLR-N) is necessary and sufficient to suppress Wnt signaling (16). However, it was still unclear whether the release of sVLDLR-N naturally occurs *in vivo* and how sVLDLR-N is generated and regulated.

The present study investigated the tissue-specific expression of the VLDLR splice variants and release of sVLDLR-N in cells and tissues. We tested the hypothesis that the VLDLR splice variants may have differential roles in regulating Wnt signaling using *VLDLR*^−/−^ mice and Wnt reporter (*Axin2*^*lacZ*^*/VLDLR*^−/−^) mice. We have further elucidated the mechanism responsible for the differential shedding rates between VLDLRI and VLDLRII and explored the functional significance of these two VLDLR variants. Most importantly, we found that VLDLR ectodomain shedding is reduced under hypoxia and in diabetes, which may contribute to the aberrant regulation of Wnt signaling in diabetic complications.

## MATERIALS AND METHODS

### Animals.

B6;129S7-*Vldlr*^*tm1Her*^/J (*VLDLR*^−/−^) mice, B6.129P2-*Axin2*^*tm1Wbm*^/J (*Axin2*^*lacZ*^) mice, BKS.Cg-*Dock7*^*m*^*+/+ Lepr*^*db*^/J (db/db) mice, C57BL/6-*Ins2*^*Akita*^ (Akita) mice, C57BLKS/J mice (as a control for db/db mice), and C57BL/6J mice (as a control for Akita mice) were obtained from the Jackson Laboratory (Bar Harbor, ME). Transgenic *Axin2*^*lacZ*^*/VLDLR*^−/−^ mice were generated at the University of Oklahoma Health Sciences Center's animal facility by crossing *Axin2*^*lacZ*^ mice with *VLDLR*^−/−^ mice, and littermate *Axin2*^*lacZ*^*/VLDLR*^*+/+*^ mice were used as control mice. Heterozygous *Axin2*^*lacZ*^ mice were used in this study. Mice were housed in a specific-pathogen-free facility and maintained in 12-h light and 12-h dark cycles. All the procedures involving mice were approved by the Institutional Animal Care and Use Committee (IACUC) at the University of Oklahoma Health Sciences Center and performed with strict adherence to the statement of the Association for Research in Vision and Ophthalmology (ARVO) for the use of animals in ophthalmic and vision research.

### Adenoviral vectors and infection of cultured cells.

Adenovirus (Ad) expressing green fluorescent protein (Ad-GFP) and adenovirus expressing VLDLRI (Ad-VLDLRI) and VLDLRII (Ad-VLDLRII) were prepared by the Gene Vector Core at the Baylor College of Medicine (18). Chinese hamster ovary (CHO) cells and cells of the ldlD cell line, a mutant CHO cell line, were separately infected with Ad-GFP, Ad-VLDLRI, and Ad-VLDLRII, which were mixed with polyethylenimine (Sigma-Aldrich, St. Louis, MO), at a multiplicity of infection (MOI) of 50 following a previously described procedure (25).

### Preparation of CM and bovine or murine IPM.

CHO, ldlD, or hTERT-RPE-1 cells (a human telomerase reverse transcriptase [hTERT]-immortalized retinal pigment epithelial [RPE] cell line) were separately infected with Ad-GFP, Ad-VLDLRI, and Ad-VLDLRII at a MOI of 50. At 48 h after infection, the culture medium was replaced with serum-free medium. Conditioned medium (CM) was then collected after 24 h of culture and centrifuged at 1,000 × *g* for 15 min at 4°C. The supernatant was collected, concentrated 4 times, and centrifuged at 100,000 × *g* for 1 h at 4°C. For hTERT-RPE-1 cells under normal culture conditions, CM was collected after 24 h of incubation of serum-free medium and centrifuged at 2,500 rpm for 15 min at 4°C. The supernatant was then collected, concentrated 20 times, and centrifuged at 100,000 × *g* for 1 h at 4°C. Bovine or murine interphotoreceptor matrix (IPM) was collected as described previously (26). Briefly, bovine retinas or mouse retinas were collected and gently rinsed with phosphate-buffered saline (PBS) (50 μl/bovine retina or 10 μl/mouse retina). The PBS was then collected and centrifuged at 1,000 × *g* for 15 min to remove cell debris, and the supernatant was passed through a 0.45-μm-pore-size syringe filter. The filtrate was collected as IPM.

### Luciferase activity assay.

A rat Müller Top-Flash cell line stably expressing a firefly luciferase gene under the control of the Wnt/β-catenin system was generated using lentivirus infection (27, 28). Wnt3A CM was obtained from L cells stably expressing human Wnt3A. Rat Müller Top-Flash cells were treated with specific CM and Wnt3A CM for 24 h, and a luciferase-based Wnt signaling activity assay (Top-Flash assay) was then conducted following the manufacturer's protocol (Promega, Madison, WI). T cell factor (TCF)/β-catenin activity was measured using a Dual-Luciferase reporter system (Promega, Madison, WI) and normalized by renilla luciferase activity.

### RT-PCR.

Total mouse RNA was extracted from murine tissues using an RNeasy minikit (Qiagen, Valencia, CA). Mouse cDNA synthesis was conducted using a cDNA synthesis kit (Applied Biosystems, Carlsbad, CA). Human heart, kidney, and retina cDNAs were purchased from the BioChain Institute (Newark, CA). Reverse transcription-PCR (RT-PCR) was performed using standard protocols and methods. The sequences of the primers used in this study are listed in Table 1.

**TABLE 1:** Primers for RT-PCR

Primer name	Primer sequence (5′–3′)
Mouse VLDLR exon 16-spanning primers Fwd	ATATCTCTGCCTGCCAGCACC
Mouse VLDLR exon 16-spanning primers Rev	TCCTCCACATCAAGTAGCCACC
Mouse HPRT1 Fwd	CAGGCCAGACTTTGTTGGAT
Mouse HPRT1 Rev	TTGCGCTCATCTTAGGCTTT
Human VLDLR exon 16-spanning primers Fwd	GGGAAAATGAAGCAGTCTATG
Human VLDLR exon 16-spanning primers Rev	GCTTTTCATGTTCTTGTGTTG
Human HPRT1 Fwd	GACCAGTCAACAGGGGACAT
Human HPRT1 Rev	CCTGACCAAGGAAAGCAAAG

### TAPI-1 and PMA treatments.

Before use, TAPI-1 (tumor necrosis factor alpha protease inhibitor 1; Peptides International, St. Louis, MO) and phorbol 12-myristate 13-acetate (PMA; Sigma-Aldrich, St. Louis, MO) were dissolved in dimethyl sulfoxide (DMSO). hTERT-RPE-1 cells were separately infected with Ad-GFP, Ad-VLDLRI, and Ad-VLDLRII at a MOI of 50. Forty-eight hours after infection, the culture medium was replaced with serum-free medium supplemented with TAPI (10 μM) or PMA (50 ng/ml) for 24 h. CM and cell lysates (CLs) were then collected.

### Western blot analysis.

Human heart, kidney, and retina proteins were purchased from the BioChain Institute (Newark, CA). Western blot analysis was performed as described previously (29). A monoclonal antibody against the N terminus of VLDLR (monoclonal antibody 3D10) was generated in a previous study (16). An antibody against the C terminus of VLDLR (antibody 6A6) was purchased from Santa Cruz (Dallas, TX). The antibodies for phosphorylated LDLR-related protein 6 (p-LRP6), nonphosphorylated β-catenin (np-β-catenin; Ser33/Ser37/Thr41), and total β-catenin (t-β-catenin) were purchased from Cell Signaling (Danvers, MA). Antibody for total-LRP6 (t-LRP6) was purchased from Santa Cruz (Dallas, TX).

### ELISA.

The plasma levels of sVLDLR-N were measured using a commercial enzyme-linked immunosorbent assay (ELISA) kit (ARP Inc., Waltham, MA). The ELISA was performed according to the manufacturer's instructions.

### Lectin staining and X-Gal staining of retinal sections.

Frozen retinal sections (8 μm) were stained with fluorescein-labeled lectin (20 μg/ml; Vector Laboratories, Burlingame, CA), and the nuclei were counter stained with DAPI (4′,6-diamidino-2-phenylindole; Vector Laboratories, Burlingame, CA). X-Gal (5-bromo-4-chloro-3-indolyl-β-d-galactopyranoside) staining of retinal sections was preformed following a documented protocol (30). Briefly, frozen retinal sections (10 μM) were cut and stained in a β-galactosidase solution [5 mM K_3_FE(CN)_6_, 5 mM K_4_Fe(CN)_6_·3H_2_O, 2 mM MgCl_2_, 0.02% NP-40, 0.1% sodium deoxycholate, 1 mg/ml 5-bromo-4-chloro-3-indolyl-β-d-galactoside] for 16 h and washed in 0.05% Tween 20 in PBS for 1 h.

### Intravitreal injection.

Intravitreal injections were performed following an established protocol (5). Adenovirus was purified using a commercial adenovirus purification kit (Clontech Laboratories, Mountain View, CA) according to the manufacturer's instructions. The mice were anesthetized with a ketamine-xylazine combination (100 mg/kg of body weight and 10 mg/kg, respectively, administered intraperitoneally). The pupils were dilated with topical application of phenylephrine (2.5% [wt/vol]) and tropicamide (1%). Purified Ad-GFP (negative-control virus), Ad-VLDLRI, and Ad-VLDLRII (1 μl/eye; 1 × 10^10^ infectious units/ml) were separately injected into the vitreous of mice at postnatal day 12 (P12) using a 34-gauge needle with an ocular injection system (WPI Inc., Sarasota, FL). Retinas or eyecups were harvested at P21 for further experiments.

### Statistical analysis.

Experiments were performed at least three times, and at least 5 mice per group were used for the animal experiments. Results from the animal studies are expressed as the mean ± standard error of the mean (SEM); data from the nonanimal studies are presented as the mean ± standard deviation (SD). Differences between groups were evaluated by a 2-tailed Student's *t* test to determine the statistical significance. Statistical significance was set at a *P* value of <0.05.

## RESULTS

### The retina expresses only VLDLRII.

**FIG 1:**
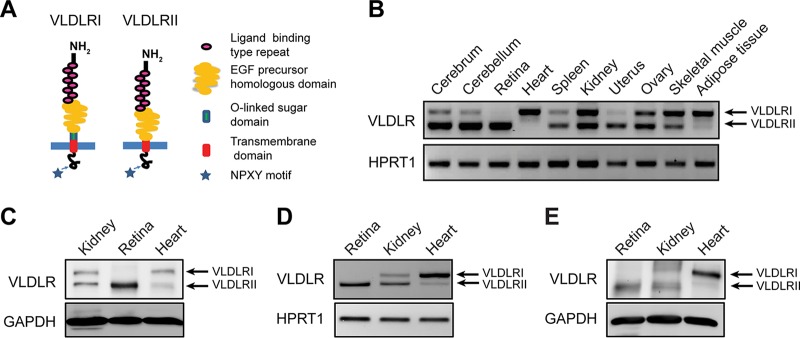
Structural difference and differential tissue distributions of VLDLR splice variants. (A) The structural difference between VLDLRI and VLDLRII is that VLDLRII lacks the O-linked sugar domain. (B) VLDLRI and VLDLRII mRNAs were detected in the indicated murine tissues by RT-PCR. As shown by agarose gel electrophoresis, the RT-PCR product from VLDLRII mRNA is 84 bp shorter than that from VLDLRI mRNA due to a lack of exon 16 in VLDLRII. (C) Expression of VLDLRI and VLDLRII in the mouse kidney, retina, and heart was measured at the protein level using Western blot analysis. (D) VLDLRI and VLDLRII mRNA levels in the human retina, kidney, and heart were measured by RT-PCR. (E) The protein levels of VLDLRI and VLDLRII in human retina, kidney, and heart were examined using Western blot analysis. EGF, endothelial growth factor; GAPDH, glyceraldehyde-3-phosphate dehydrogenase; HPRT1, hypoxanthine phosphoribosyltransferase 1.

It is known that the structural difference between VLDLRI and VLDLRII is a lack of the O-linked sugar domain in VLDLRII (Fig. 1A). To understand the biological roles of the VLDLR splice variants, we first studied the tissue-specific expression patterns of VLDLRI and VLDLRII. As shown by RT-PCR using primers spanning the sequence encoding the O-linked sugar domain, the VLDLRI mRNA was abundantly expressed in the murine heart, adipose tissue, and skeletal muscle, while the VLDLRII mRNA was highly expressed in the murine brain, retina, spleen, kidney, ovary, and uterus (Fig. 1B). Interestingly, among the examined tissues, muscular tissues, such as heart and skeletal muscle tissues, mainly expressed VLDLRI, while the retina exclusively expressed VLDLRII, as shown at both the mRNA and protein levels (Fig. 1B and C). The expression of the VLDLR splice variants in the human retina, kidney, and heart was further verified. Consistent with the results from the murine tissues, the human heart mainly expressed VLDLRI, while the human retina expressed only VLDLRII at both the mRNA and protein levels (Fig. 1D and E).

### Presence of sVLDLR-N in CM from RPE cells and IPM.

**FIG 2:**
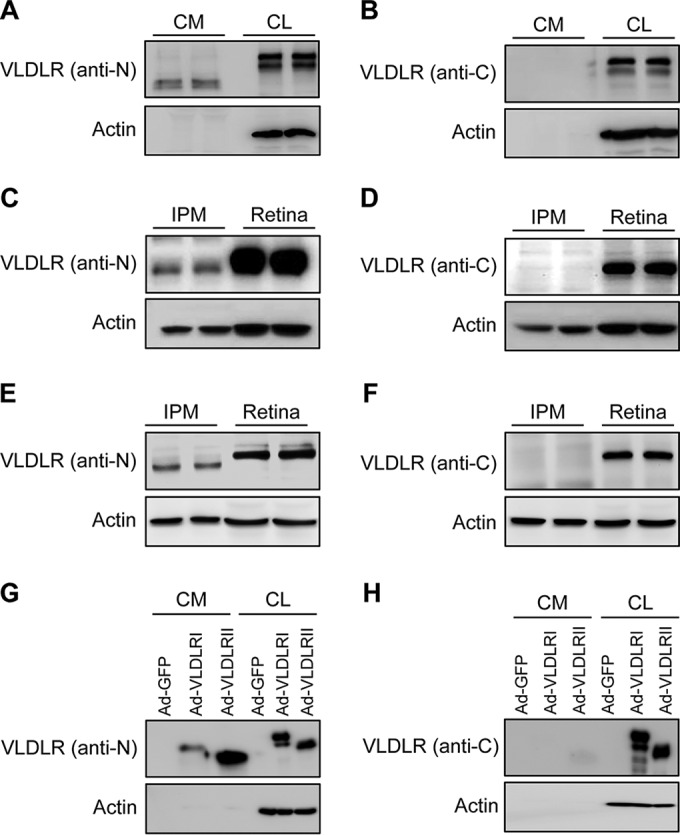
Presence of sVLDLR-N in CM from RPE cells, IPM from retinas, and CM from cells expressing VLDLR. CM and total CLs were collected from hTERT-RPE-1 cells. (A and B) sVLDLR-N levels in CM and VLDLR in CLs were measured separately by Western blotting using an antibody against the N terminus of VLDLR (anti-N; antibody 3D10) (A) and an antibody against the C terminus of VLDLR (anti-C; antibody 6A6) (B), respectively. (C and D) sVLDLR-N levels in bovine IPM and VLDLR levels in bovine retinas were measured by Western blotting using antibodies 3D10 (C) and 6A6 (D), respectively. (E and F) Similarly, sVLDLR-N in murine IPM and VLDLR in murine retinas were detected using antibodies 3D10 (E) and 6A6 (F), respectively. (G and H) CHO cells were separately infected with Ad-GFP, Ad-VLDLRI, or Ad-VLDLRII at a MOI of 50. sVLDLR-N in CM and VLDLR in CLs were separately measured by Western blotting using antibodies 3D10 (G) and 6A6 (H), respectively.

Previous studies indicated that recombinant VLDLR was subjected to extracellular domain cleavage (24, 31). However, the endogenous shed soluble VLDLR extracellular domain (sVLDLR-N) was not found in body fluids or tissues. Thus, we determined whether ectodomain shedding of VLDLR is a naturally occurring biological process in cells or retinas. Using antibodies against the N terminus of VLDLR, Western blot analysis showed that cells of the hTERT-RPE-1 cell line released sVLDLR-N into CM under normal culture conditions (Fig. 2A). In addition, sVLDLR-N was also detected in both the bovine interphotoreceptor matrix (IPM) (Fig. 2C) and the murine IPM from the retinas (Fig. 2E). The presence of sVLDLR-N in CM and IPM was identified by its molecular weight, which was lower than that of uncleaved full-length VLDLR (FL-VLDLR) in cell lysates (CLs) (Fig. 2A) and in IPM from the retinas (Fig. 2C and E), respectively, and by its lack of the intracellular domain, as shown by Western blotting with antibodies for the C terminus of VLDLR (Fig. 2B, D, and F). Further, CHO cells infected with adenoviral vectors expressing VLDLRI or VLDLRII released sVLDLR-N into CM (Fig. 2G and H), indicating that both VLDLRI and VLDLRII undergo ectodomain shedding.

### Higher ectodomain shedding rate and more potent Wnt inhibitory effects of VLDLRII *in vitro*.

**FIG 3:**
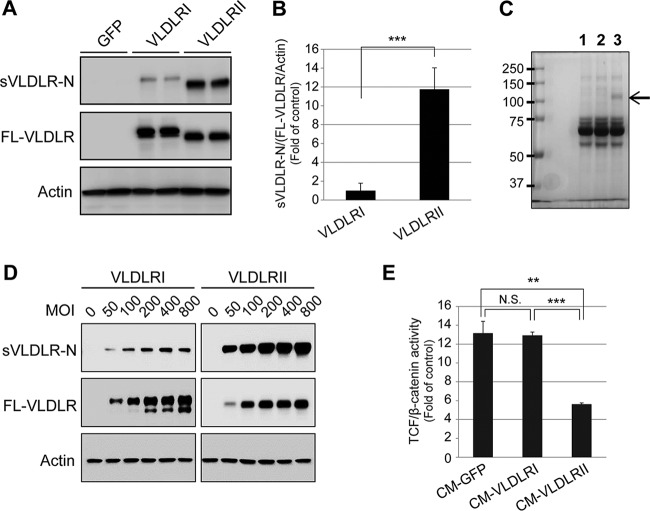
A higher ectodomain shedding rate and a more potent Wnt pathway inhibitory effect of VLDLRII than VLDLRI *in vitro*. (A) CM and CLs were collected from CHO cells expressing GFP, VLDLRI, or VLDLRII. sVLDLR-N in CM and full-length VLDLR (FL-VLDLR) in CLs were detected using the 3D10 antibody. (B) The levels of the proteins were semiquantified by densitometry, and the ratios of sVLDLR-N to FL-VLDLR (normalized by the β-actin levels) were calculated and compared between VLDLRI and VLDLRII. (C) The total proteins in CM from cells expressing GFP (lane 1), VLDLRI (lane 2), or VLDLRII (lane 3) were analyzed by SDS-PAGE and stained by Coomassie blue. The band indicated by an arrow had a molecular weight identical to that of sVLDLR-N and existed only in CM from cells expressing VLDLRII. (D) CHO cells were separately infected with Ad-GFP, Ad-VLDLRI, or Ad-VLDLRII at the indicated MOIs. Similarly, sVLDLR-N and FL-VLDLR levels were measured by Western blotting. (E) Müller Top-Flash cells were treated with CM from CHO cells expressing VLDLRI or VLDLRII and Wnt3A CM for 16 h. The cells were then lysed, and TCF/β-catenin transcriptional activity was measured and normalized to that of control CM from cells expressing GFP. All data are representative of those from 3 independent experiments. Values are means ± SDs. *P* values were determined by Student's *t* test for two-group comparisons. **, *P* < 0.01; ***, *P* < 0.001; N.S., nonsignificant.

Next, we investigated whether the VLDLR splice variants had differential ectodomain shedding rates. VLDLRI and VLDLRII were separately expressed in CHO cells using adenoviral vectors. The protein levels of sVLDLR-N in CM and FL-VLDLR in CLs were measured. The levels of sVLDLR-N were significantly higher in CM from cells expressing VLDLRII than in CM from cells expressing VLDLRI, while the levels of FL-VLDLRI and FL-VLDLRII in CLs were similar (Fig. 3A). In addition, the ratios of sVLDLR-N to FL-VLDLR (normalized by β-actin levels) were calculated and compared between VLDLRI and VLDLRII, which indicated that VLDLRII has a significantly higher ectodomain shedding rate than VLDLRI (Fig. 3B). Moreover, Coomassie blue staining of total proteins in CM from cells expressing GFP, VLDLRI, or VLDLRII further confirmed that CM from cells expressing VLDLRII contained a higher level of sVLDLR-N (Fig. 3C). Additionally, the levels of sVLDLR-N in CM correlated with the expression of FL-VLDLR in CLs (Fig. 3D).

Our previous studies have shown that recombinant sVLDLR-N is essential and sufficient for Wnt signaling inhibition (16). We hypothesized that CM containing sVLDLR-N from cells expressing the VLDLR splice variants may have differential inhibitory effects on Wnt signaling, as they have differential ectodomain shedding rates. As demonstrated by the Top-Flash assay, CM from cells expressing VLDLRII exhibited a more potent inhibitory effect on Wnt signaling than that from cells expressing VLDLRI (Fig. 3E), which correlated with the levels of sVLDLR-N shed into CM. In contrast, no inhibitory effect was detected in control CM from cells expressing GFP (Fig. 3E).

### More potent Wnt signaling inhibitory effects of VLDLRII *in vivo*.

**FIG 4:**
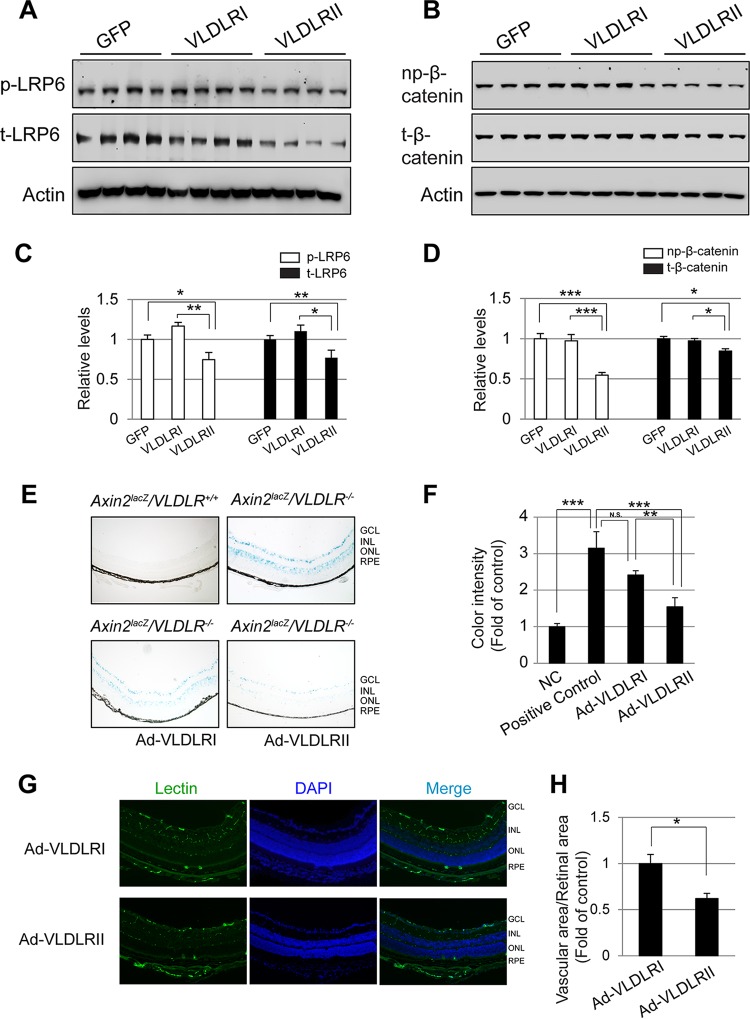
VLDLRII has a more potent inhibitory effect on Wnt signaling than VLDLRI *in vivo*. (A and B) The levels of phosphorylated LRP6 (p-LRP6) and total LPR6 (t-LRP6) (A) and the levels of nonphosphorylated β-catenin (np-β-catenin) and total β-catenin (t-β-catenin) (B) in the eyecups of *VLDLR*^−/−^ mice intravitreally injected with Ad-GFP, Ad-VLDLRI, or VLDLRII were measured by Western blotting. (C and D) Densitometry was performed to semiquantify p-LPR6 and t-LRP6 (C) and np-β-catenin and t-β-catenin (D), the levels of which were normalized by the β-actin levels. (E) Wnt signaling activity in the retina was evaluated by X-Gal staining of retinal sections from *Axin2*^*lacZ*^*/VLDLR*^*+/+*^ (negative control), *Axin2*^*lacZ*^*/VLDLR*^−/−^ (positive control), and *Axin2*^*lacZ*^*/VLDLR*^−/−^ mice injected with adenovirus expressing VLDLRI (Ad-VLDLRI) and *Axin2*^*lacZ*^*/VLDLR*^−/−^ mice injected with adenovirus expressing VLDLRII (Ad-VLDLRII). GCL, ganglion cell layer; INL, inner nuclear layer; ONL, outer nuclear layer; RPE, retinal pigment epithelium. Magnifications, ×100. (F) The color intensities of the retinal section images were quantified and compared among the four groups shown in panel E. (G) Vascular endothelial cells in the retinal sections of *VLDLR*^−/−^ mice injected with Ad-VLDLRI or Ad-VLDLRII were stained with lectin (green). The nuclei were counterstained with DAPI (blue). Magnifications, ×200. (H) The vascular areas (lectin staining shown in panel G) in the retinal sections were calculated and compared between *VLDLR*^−/−^ mice injected with Ad-VLDLRI or Ad-VLDLRII. All data are representative of those from 3 independent experiments with 5 to 8 mice per group. Values are means ± SEMs. *P* values were determined by Student's *t* test. *, *P* < 0.05; **, *P* < 0.01; ***, *P* < 0.001; N.S., nonsignificant.

To verify the differential Wnt signaling inhibitory effects of VLDLRI and VLDLRII *in vivo*, we separately injected Ad-VLDLRI, Ad-VLDLRII, and control Ad-GFP into the vitreous of *VLDLR*^−/−^ mice, which are known to have overactivated Wnt signaling in the retina (11). The levels of Wnt signaling components p-LRP6, t-LRP6, np-β-catenin, and t-β-catenin were significantly decreased in the eyecups of *VLDLR*^−/−^ mice injected with Ad-VLDLRII but not in those of mice injected with Ad-VLDLRI or Ad-GFP (Fig. 4A to D). These results illustrate that VLDLRII, but not VLDLRI, can significantly decrease Wnt signaling activity in the retinas of *VLDLR*^−/−^ mice, suggesting that the VLDLR splice variants have differential roles in Wnt signaling regulation *in vivo*.

The *Axin2*^*lacZ*^ mouse, a Wnt signaling reporter mouse (32), was utilized to further confirm the role of the VLDLR splice variants in Wnt signaling regulation. To exclude the possibility of the influence of endogenous VLDLR, we generated *Axin2*^*lacZ*^*/VLDLR*^−/−^ mice by crossing the *Axin2*^*lacZ*^ mouse with the *VLDLR*^−/−^ mouse. As shown by X-Gal staining of retinal sections, *Axin2*^*lacZ*^*/VLDLR*^−/−^ mice displayed upregulated Wnt signaling activity in the retina, mainly in the ganglion cell layer and inner nuclear layer, compared with the level of regulation in *Axin2*^*lacZ*^*/VLDLR*^*+/+*^ control mice (Fig. 4E). This confirmed our previous observation that Wnt signaling is upregulated in the retina of *VLDLR*^−/−^ mice (11). In addition, injection of Ad-VLDLRII significantly decreased the Wnt signaling-mediated β-galactosidase activity in the retina of *Axin2*^*lacZ*^*/VLDLR*^−/−^ mice, while injection of Ad-VLDLRI had less of an effect on β-galactosidase activity (Fig. 4E and F), confirming that VLDLRII has a more potent inhibitory effect on Wnt signaling than VLDLRI in the retina. Further, lectin staining of retinal sections showed that the retinal vascular density was lower in *VLDLR*^−/−^ mice injected with Ad-VLDLRII than in mice injected with Ad-VLDLRI, suggesting that VLDLRII has a more potent antiangiogenic effect (Fig. 4G and H), consistent with its suppression of Wnt signaling.

### Roles of O-glycosylation in ectodomain shedding of VLDLRI and VLDLRII.

**FIG 5:**
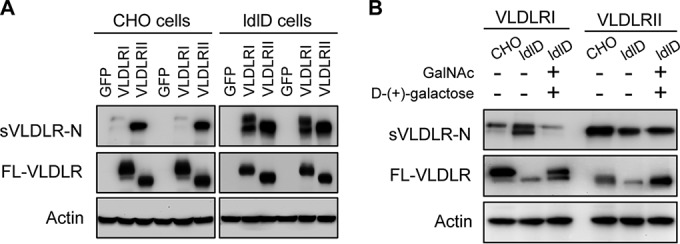
Roles of O-glycosylation in ectodomain shedding of VLDLRI and VLDLRII. (A) CM and CLs were separately collected from WT CHO cells expressing VLDLRI or VLDLRII and O-glycosylation-deficient ldlD cells expressing VLDLRI or VLDLRII. sVLDLR-N and FL-VLDLR were measured with the 3D10 antibody. The levels of sVLDLR-N in CM from ldlD cells expressing VLDLRI were dramatically increased compared with those in CM from WT CHO cells expressing VLDLRI. (B) CM and CLs were separately collected from WT CHO cells expressing VLDLRI or VLDLRII and ldlD cells expressing VLDLRI or VLDLRII in the presence (lanes +) or absence (lanes −) of 10 μM d-(+)-galactose and 100 μM *N*-acetyl-d-galactosamine (GalNAc). sVLDLR-N and FL-VLDLR were measured with the 3D10 antibody. In the presence of d-(+)-galactose and GalNAc, VLDLRI and VLDLRII showed patterns of ectodomain shedding in ldlD cells similar to that in WT CHO cells.

It was reported that most of the mucin-type serine/threonine-linked (O-linked) oligosaccharides in VLDLR are attached to the O-linked sugar domain (24). To determine the role of O-glycosylation in ectodomain shedding of the VLDLR splice variants, VLDLRI and VLDLRII were separately expressed in wild-type (WT) CHO cells and cells of the ldlD cell line, a mutant CHO cell line with a reversible defect in protein O-glycosylation (33), and the levels of sVLDLR-N and FL-VLDLR were measured. While the levels of full-length VLDLRI and VLDLRII in both WT CHO and ldlD cells were similar, the levels of sVLDLR-N in CM from ldlD cells expressing VLDLRI were dramatically increased compared with those in CM from WT CHO cells expressing VLDLRI (Fig. 5A). In contrast, the levels of sVLDLR-N in CM from WT CHO cells expressing VLDLRII were similar to those in CM from ldlD cells expressing VLDLRII (Fig. 5A). These results suggest that O-glycosylation is important for the stability of VLDLRI and responsible for the lower ectodomain shedding rate of VLDLRI relative to that of VLDLRII.

To further verify the roles of O-glycosylation in VLDLR ectodomain shedding, we restored protein O-glycosylation in ldlD cells by exposing the cells to d-(+)-galactose and *N*-acetyl-d-galactosamine (GalNAc), which cannot be synthesized efficiently in ldlD cells due to the defects of the enzyme UDP-galactose and UDP-*N*-acetylgalactosamine 4-epimerase (33). As shown by Western blotting, in the presence of d-(+)-galactose and GalNAc, VLDLRI and VLDLRII in ldlD cells showed patterns of ectodomain shedding similar to those in WT CHO cells, demonstrating that restored O-glycosylation in ldlD cells neutralized the effects of O-glycosylation deficiency on VLDLRI ectodomain shedding (Fig. 5B). Taken together, these results suggested that O-glycosylation determines the ectodomain shedding rates of VLDLRI and VLDLRII.

### VLDLR ectodomain shedding inhibited by TAPI-1 and promoted by PMA.

**FIG 6:**
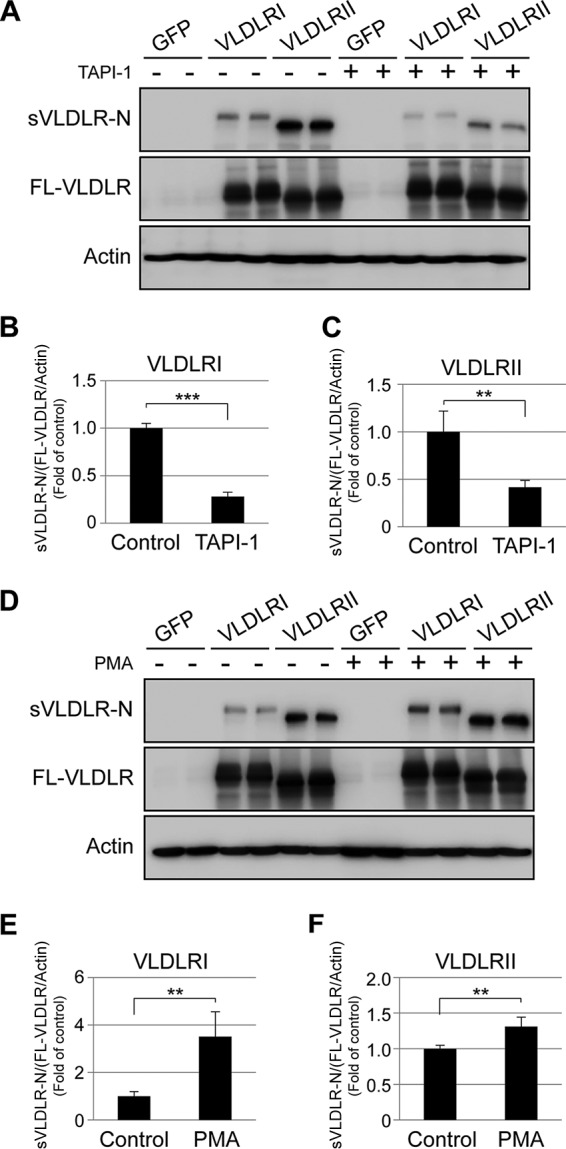
TAPI-1 inhibits while PMA promotes the release of sVLDLR-N. (A) hTERT-RPE-1 cells expressing GFP, VLDLRI, or VLDLRII were treated with TAPI-1 (10 μM) or DMSO (control) for 24 h. CM and CLs were then collected. sVLDLR-N and FL-VLDLR were measured by Western blotting with the 3D10 antibody. (B and C) Levels of proteins were semiquantified by densitometry, and the ratios of sVLDLR-N to FL-VLDLR (normalized by the β-actin level) in cells expressing VLDLRI (B) and VLDRII (C) were calculated and compared with those in the groups treated with the control. (D) Similarly, cells expressing GFP, VLDLRI, or VLDLRII were treated with PMA (50 ng/ml). sVLDLR-N and FL-VLDLR were measured by Western blotting with the 3D10 antibody. (E and F) The ratios of sVLDLR-N to FL-VLDLR (normalized by the β-actin level) in cells expressing VLDLRI (E) and VLDRII (F) were calculated and compared with those in groups treated with the control. Data are representative of those from 3 independent experiments. Values are means ± SDs. *P* values were determined by Student's *t* test. **, *P* < 0.01; ***, *P* < 0.001.

To further understand the shedding process of the VLDLR extracellular domain, TAPI-1, a matrix metalloproteinase (MMP) inhibitor, and PMA, a protein kinase C (PKC) activator, were selected to treat RPE cells expressing VLDLRI and VLDLRII. As shown by Western blotting, TAPI-1 decreased the ectodomain shedding rates of VLDLRI and VLDLRII to approximately 28% and 42%, respectively, of those for the control groups (Fig. 6A to C). This suggests that MMPs may be responsible for the extracellular domain cleavage of VLDLR. In contrast, PMA increased the shedding rate of VLDLRI by 2.5-fold and that of VLDLRII by 1.31-fold relative to the rate for the control groups treated with vehicle (Fig. 6D to F), suggesting that PKC promotes ectodomain shedding of VLDLR.

### Suppression of sVLDLR-N release by hypoxia.

**FIG 7:**
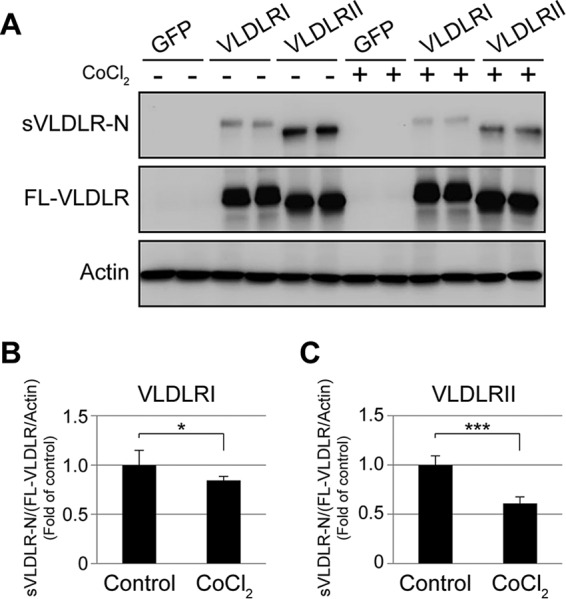
Suppressed sVLDLR-N release under hypoxia. (A) hTERT-RPE-1 cells were separately infected with Ad-GFP, Ad-VLDLRI, or Ad-VLDLRII. After 48 h, the culture medium was replaced with serum-free medium, and the cells were treated with 200 μM CoCl_2_ for another 24 h. CM and CLs were then collected. sVLDLR-N and FL-VLDLR were measured by Western blotting with the 3D10 antibody. (B and C) The levels of the proteins were semiquantified by densitometry, and the ratios of sVLDLR-N to FL-VLDLR (normalized by the β-actin level) were calculated and compared between the control group and the group treated with CoCl_2_ in cells expressing VLDLRI (B) and VLDLRII (C). Data are representative of those from 3 independent experiments. Values are means ± SDs. *P* values were determined by Student's *t* test. *, *P* < 0.05; ***, *P* < 0.001.

To understand the regulation of VLDLR ectodomain shedding under stress conditions, we investigated whether the sVLDLR-N release was affected by hypoxia, a common stressor in diabetic complications. CHO cells expressing VLDLRI and VLDLRII were exposed to CoCl_2_, a commonly used inducer of hypoxia in cultured cells (34). Hypoxia decreased the shedding rates of VLDLRI and VLDLRII to ∼84% and ∼64%, respectively, compared with the rate for the normoxia control groups (Fig. 7). These results indicate that the release of sVLDLR-N was suppressed under hypoxia.

### Decreased plasma levels of sVLDLR-N in type 1 and type 2 diabetic animal models.

**FIG 8:**
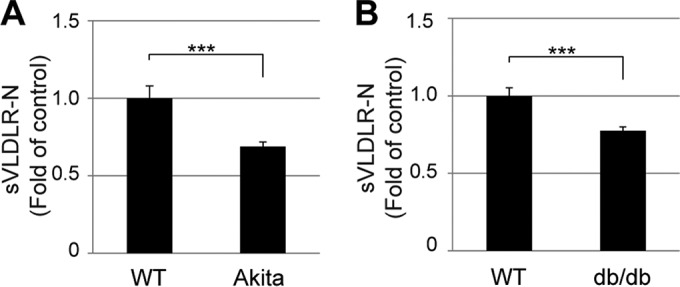
Decreased levels of sVLDLR-N in the plasma of type 1 and type 2 diabetic mouse models. The sVLDLR-N level was separately measured in the plasma of 3-month-old Akita mice and age-matched control mice (A) and 6-month-old db/db mice and age-matched nondiabetic control mice (B) using an ELISA kit with a capture antibody against the N terminus of VLDLR. The levels of sVLDLR-N in plasma are expressed as the fold change from those in nondiabetic control mice. Values indicate means ± SEMs for 8 mice per group. *P* values were determined by Student's *t* test. ***, *P* < 0.001.

Previous studies have shown that chronic hypoxia plays a pathogenic role in diabetic complications, such as diabetic retinopathy and diabetic cardiomyopathy (35, 36). We hypothesized that the levels of sVLDLR-N may be reduced in diabetic animals, as hypoxia suppresses sVLDLR-N release. To test this hypothesis, plasma levels of sVLDLR-N were measured in two commonly used diabetic animal models: Akita mice, a type 1 diabetes (T1D) model (37, 38), and db/db mice, a type 2 diabetes (T2D) model (39), as well as their age- and genetic background-matched nondiabetic controls. As demonstrated by ELISA, plasma levels of sVLDLR-N were significantly reduced in Akita and db/db mice in comparison to those in their respective WT controls (∼69% and ∼77% of control levels, respectively) (Fig. 8), suggesting that the decline in the level of sVLDLR-N shedding may be associated with diabetic complications.

## DISCUSSION

The dysregulation of Wnt signaling plays pathological roles in a number of diseases, such as cancer, cardiomyopathy, diabetic nephropathy, and diabetic retinopathy (1, 2, 4, 5). Elucidation of Wnt signaling regulation is of great significance for understanding these diseases and discovering new therapeutic targets. Our previous studies have demonstrated that VLDLR functions as a negative regulator of Wnt signaling. In the present study, we first showed that the two major VLDLR splice variants have differential activities in regulating Wnt signaling due to their different ectodomain shedding rates, which identified the functional difference of these splice variants. The regulation of Wnt signaling by VLDLR was originally thought to be limited intracellularly, as VLDLR is a transmembrane protein. However, the present study demonstrates for the first time that VLDLR ectodomain shedding occurs endogenously and that released sVLDLR-N can function as a soluble endogenous peptide to modulate Wnt signaling intercellularly, suggesting a novel mechanism for Wnt signaling regulation. Meanwhile, the present study demonstrated that the differential ectodomain shedding rates of VLDLRI and VLDLRII are ascribed to the lack of the O-linked sugar domain in VLDLRII. Further, the present study identified that ectodomain shedding of VLDLR is suppressed by hypoxia and in diabetes, which may contribute to the overactivation of Wnt signaling in diabetic complications. Together, these observations reveal a new mechanism for Wnt signaling regulation and provide new insights into diseases associated with abnormal Wnt signaling.

The tissue-specific expression patterns of VLDLRI and VLDLRII have been previously reported (17) and further confirmed in our current study. However, the biological significance of these specific expression patterns was previously unknown. Our study demonstrated for the first time that the retina expresses only VLDLRII and the heart expresses mainly VLDLRI in both the mouse and human. In addition, several studies have indicated that lack of VLDLR promotes angiogenesis and induces inflammatory responses in the retina, indicating the critical roles of VLDLR in the regulation of normal retinal vascularization and retinal angiogenic diseases (5, 13–15). Moreover, studies have shown that VLDLRI has a higher affinity for β-VLDL than VLDLRII and therefore may play a major role in lipoprotein uptake and metabolism (17). In contrast, our study demonstrated that VLDLRII has a more potent inhibitory effect on Wnt signaling than VLDLRI. Together, these observations suggest that VLDLRI is likely to mainly function as a membrane receptor for lipid metabolism, while VLDLRII may play a major role in other biological functions beyond lipid metabolism, such as Wnt signaling regulation. It remains to be investigated how the tissue-specific alternative splicing of the *VLDLR* gene is regulated.

Recent studies have also shown that VLDLRII undergoes more rapid extracellular domain shedding than VLDLRI in a forced expression system (24). Another study showed that a fragment of VLDLR shed from HeLa cells inhibited human rhinovirus infection (31). In the present study, we have shown that VLDLRII has a higher extracellular shedding rate than VLDLRI. Moreover, we have also demonstrated that shedding of sVLDLR-N occurs in both recombinant VLDLR and endogenous VLDLR *in vitro*. sVLDLR-N was also found in the extracellular matrix of bovine and murine retinas (Fig. 2), in mouse plasma (Fig. 8), and in human plasma (data not shown). This provided the first evidence that the release of sVLDLR-N is a naturally occurring biological process. Further, VLDLRII exhibited higher Wnt inhibitory effects than VLDLRI by more efficiently releasing sVLDLR-N. Together, sVLDLR-N may function as a soluble endogenous modulator of Wnt signaling to exert intercellular regulatory effects.

To study the physiological significance of our findings, we utilized two animal models, *VLDLR*^−/−^ mice and Wnt reporter (*Axin2*^*lacZ*^*/VLDLR*^−/−^) mice. Ablation of VLDLR upregulates Wnt signaling in the retina (11). Using VLDLRI and VLDLRII gene delivery, we found that VLDLRII, but not VLDLRI, inhibited Wnt signaling activity in the retina. The inhibition of Wnt signaling results in suppression of retinal neovascularization in mice injected with VLDLRII, as Wnt signaling regulates pathological vascular growth, as reported by many studies (5, 11, 40). The differential functions of the VLDLR splice variants may be attributed to the differential rates of shedding of the VLDLR extracellular domain, as CM from cells expressing VLDLRII exhibited a more potent Wnt inhibitory effect than that from cells expressing VLDLRI. This conclusion is also supported by our recent study showing that recombinant sVLDLR-N can inhibit Wnt signaling and ocular neovascularization (30). We also found that ablation of VLDLR in the Wnt reporter mice upregulated Wnt signaling in the inner retina, further supporting our hypothesis that sVLDLR-N is released and exerts an intercellular Wnt regulatory effect, as VLDLR is reported to be highly expressed in the photoreceptor layer and RPE and not in the inner retina (41).

O-glycosylation in VLDLRI is likely to serve as a barrier to enzymes that catalyze the extracellular domain shedding, since the O-glycosylation domain of VLDLRI is located close to the cell membrane and attached by numerous carbohydrate side chains. To verify the significance of glycosylation in VLDLR ectodomain shedding, we used cells of the ldlD cell line, a mutant CHO cell line deficient in O-glycosylation. In the O-glycosylation-deficient cells, VLDLRI and VLDLRII exhibited similar ectodomain shedding rates, while they showed differential ectodomain shedding rates in WT CHO cells. Furthermore, restoration of O-glycosylation in ldlD cells led to differential ectodomain shedding rates between VLDLRI and VLDLRII, similar to the findings for WT CHO cells. These results suggest that O-glycosylation plays a key role in determining the ectodomain shedding rates of VLDLRI and VLDLRII, which is consistent with previous reports (24). We also investigated the regulation of ectodomain shedding of VLDLR by treating cells expressing VLDLRI or VLDLRII with TAPI-1 and PMA. TAPI-1 is commonly used as a general MMP inhibitor (42), while PMA is a PKC activator and induces ectodomain shedding of many transmembrane proteins, such as amyloid precursor protein (APP), IACM-1, and l-selectin (43–45). Here, we demonstrated that ectodomain shedding of VLDLR was inhibited by TAPI-1 and induced by PMA. These observations indicate that MMPs may be responsible for the ectodomain shedding of VLDLR. Further studies are warranted to identify specific MMPs that cleave the extracellular domain of VLDLR.

Hypoxia is known to induce activation of Wnt signaling in mouse embryonic cells and the hippocampus of adult mice (46, 47). Interestingly, our results demonstrated that the release of sVLDLR-N is inhibited under hypoxia. As sVLDLR-N inhibits Wnt signaling, the reduced release of sVLDLR-N under hypoxia may be responsible for the hypoxia-induced upregulation of Wnt signaling.

Abnormal Wnt signaling activation has been reported in endothelial dysfunction and diabetic vascular complications, such as diabetic retinopathy and diabetic nephropathy (5, 6). Here, we used Akita mice and db/db mice to investigate the possible roles of sVLDLR-N in early stages of diabetic complications. Akita mice develop T1D at 1 month of age, and by 3 months they have been shown to exhibit early features of diabetic retinopathy, such as increased retinal blood flow and increased acellular capillaries (48). db/db mice, a commonly used T2D mouse model, have elevated blood glucose levels at the age of 2 months. Six-month-old db/db mice have been reported to develop nonproliferative diabetic retinopathy, such as endothelial inflammation and vascular leakage (49, 50). Interestingly, the levels of circulating sVLDLR-N were decreased in both 3-month-old Akita mice and 6-month-old db/db mice, suggesting reduced VLDLR ectodomain shedding under diabetes conditions. As VLDLR is highly expressed in endothelial cells (51), it is possible that reduced VLDLR shedding results in the dysregulation of Wnt signaling, leading to endothelial cell dysfunction in diabetic complications. Further studies will be focused on determining if sVLDLR-N in the circulation can be used as a biomarker for the prognosis and diagnosis of diabetic complications.
